# The ICPO Foundation—on the way toward collaborative growth of theranostics

**DOI:** 10.1007/s00259-021-05651-2

**Published:** 2022-01-05

**Authors:** Richard P. Baum, Josh Mailman, Odile Jaume

**Affiliations:** 1 Curanosticum Wiesbaden-Frankfurt, Wiesbaden, Germany; 2 NorCal CarciNET Community, San José, CA USA; 3 The ICPO Foundation, Humboldtstr. 29, 65189, Wiesbaden, Germany

Today, the paradigm shift in cancer care to Precision Oncology is well recognized. In this shift, the Theranostics Community needs to live up to its potential by demonstrating its ability to improve the current standard of care: from late to early diagnosis, from bystander to being actively involved in patient care, from severe side effects to minimal side effects, from exploding costs to sustainable health economics, from months to years of prolonged survival, and from low quality of life to preserved and improved quality of life.

There is still room to grow on the path toward expanding international patient access to the theranostics toolbox, including improving standardization of clinical practices and certified professional training. It is encouraging to see that rising radiopharmaceutical forecasts presented by the SNMMI and EANM are indicating an increase in the number of radiopharmaceutical therapies performed. The question is, thus, how to support this growth, and how to collaborate and ensure the expected patient outcomes prove their value?

In 2019, the International Centers for Precision Oncology Foundation (ICPO) was established with the aim to answer this question. Our goal is to unleash the potential of theranostics for international patients, regardless of their backgrounds and conditions, via improved access to certified education curriculum and standardized infrastructure models for those providing theranostics. The ICPO Founders, namely Prof. Dr. Richard P. Baum, Udo J. Vetter, Michael Lee-Chin, and Oliver Buck, strongly believe that a collaborative approach is the only way forward to growing theranostics. Therefore, the ICPO mission is to help scale patient access together through education, know-how transfer, and standardization in clinical practice via creating the ICPO Community, Academy, and Center « Social Franchise» network at a global level.

As a call for action to the broad Theranostics Community and ICPO members, the ICPO Foundation held, with great success, its “Second ICPO FORUM for Theranostics in Precision Oncology” on October 18–19, 2021, in Garching (Munich), Germany. On this occasion, 210 medical professionals from around the globe joined in and fostered a shared and future-oriented vision for a self-amplifying growth of theranostics. Moreover, 22 world-class speakers and 17 industry sponsors supported this hybrid event and contributed to content and visibility despite the pandemic context.

Among the ICPO Forum, highlights were talks by patients and nurses, urging for more transparency and multidisciplinary approaches so patients can be more involved and informed about their therapy while reassured about safety, efficacy, quality of life, and discrepancies across geographies and clinical centers. Moreover, the need for standardization of care, hand-in-hand with education and center certifications, including audits, was also emphasized in the ICPO talks and relayed by the International Atomic Energy Agency representative during the panel discussion.

Over the last year, the ICPO Academy—structured around e-learning and hands-on courses/practical training—has been developed thanks to a public–private partnership (PPP) supported by the German International Cooperation Society (Gesellschaft für Internationale Zusammenarbei, GIZ). The first pilot phase will involve five Chinese hospitals. ICPO’s primary aim is to allow more physicians, radiochemists, physicists, and nurses/technologists to receive certified theranostics training worldwide. Secondly, ICPO will introduce the concept of ICPO Centers, which will strategically spearhead standardizing, certifying, and scaling patient access for Radiomolecular Precision Oncology (RPO) worldwide, essentially building a «social franchise model» for dedicated and optimized Precision Oncology Centers. Lastly, ICPO supports Scientific Research from all theranostics perspectives, ranging from radiopharmaceutical development to artificial intelligence, image-guided medicine, or virtual reality models, thanks to the 15 international members of the Scientific Advisory Board.

Embracing a community responsibility, the ICPO Foundation today encourages all leaders within the Nuclear Medicine Community as well as related pharmaceutical, industrial, medical, financial, and philanthropic sectors, who recognize the current paradigm shift in cancer care, to champion the ICPO cause by joining us as members of our community. Together we can initiate projects with a higher sense of purpose for oncology patients worldwide. We hope that Radiomolecular Precision Oncology lives up to its promise, curative and abundant to all patients in need irrespectively of race, country, social status, and all else.

